# Ovarian metastases from ileum cancer in a patient with germline *EPCAM* gene deletion successfully treated with surgical resection and CAPOX chemotherapy: a case report

**DOI:** 10.1186/s12881-020-01013-1

**Published:** 2020-04-09

**Authors:** Narushi Iwata, Ayumi Shikama, Wataru Takao, Yoshihiko Hosokawa, Hiroya Itagaki, Nobutaka Tasaka, Azusa Akiyama, Hiroyuki Ochi, Takeo Minaguchi, Miwa Arita, Emiko Noguchi, Toshikazu Moriwaki, Toyomi Satoh

**Affiliations:** 1 Department of Obstetrics and Gynecology, Faculty of Medicine, University of Tsukuba, 1-1-1 Tennodai, Tsukuba, Ibaraki, 305-8575 Japan; 2 Department of Medical Genetics, Faculty of Medicine, University of Tsukuba, Tsukuba, Japan; 3 Department of Gastroenterology, Faculty of Medicine, University of Tsukuba, Tsukuba, Japan

**Keywords:** Lynch syndrome, *EPCAM*, Ileum cancer, Ovarian metastases

## Abstract

**Background:**

Despite recent findings that epithelial cell adhesion molecule (*EPCAM*) deletions can cause Lynch syndrome (LS), its clinical characteristics are still unknown. We present the first case of ileum cancer in a patient with germline *EPCAM* gene deletion, which was discovered during ovarian tumor surgery.

**Case presentation:**

A 59-year-old woman presented with a history of colon cancer occurring at 38 and 55 years old. Five of her siblings had a history of colon cancer, and an elder sister had confirmed LS. As imaging examination revealed an ovarian tumor, and we performed hysterectomy and bilateral salpingo-oophorectomy. Careful observation during surgery revealed a cherry-sized tumor in the ileum, prompting partial ileal resection. Pathological examination showed the ovarian tumor to be a metastasis of ileum cancer. Genetic testing with blood-relative information using multiplex ligation-dependent probe amplification showed *EPCAM* exons 8 and 9 deletions, confirming LS. The patient received adjuvant chemotherapy with CAPOX (capecitabine and oxaliplatin) and has remained disease-free for 24 months.

**Conclusions:**

We were fortunate to identify ileum cancer that would have been difficult to find preoperatively through careful observation during ovarian tumor surgery and successfully treated the patient by using surgical resection and CAPOX chemotherapy. When treating patients with hereditary cancer syndromes including LS, we should keep all associated cancers in mind.

## Background

Lynch syndrome (LS) is caused by pathogenic germline variants in DNA mismatch repair (MMR) genes, *MLH1*, *MSH2*, *MSH6*, and *PMS2*, and leads to a high risk of colorectal, endometrial, and several other extra-colonic cancers [1]. Recently, germline deletions in the epithelial cell adhesion molecule gene (*EPCAM*), which lies upstream of *MSH2*, have been identified as a novel cause of LS. Deletion of the 3′ region of *EPCAM* leads to subsequent epigenetic silencing of the *MSH2* promoter region, resulting in MMR deficiency [2, 3].

*EPCAM* deletions account for about 1–3% of all pathogenic variants in LS [4]. The risk of colorectal cancer in individuals with *EPCAM* deletions is comparable to those with MMR pathogenic variants in LS, whereas the cumulative risk of extra-colonic cancer is much lower compared to those with MMR pathogenic variants in LS [5]. However, there are few reports about LS patients with *EPCAM* deletions, and their clinical characteristics are still unknown.

Here, we present the first case of ileum cancer in a patient with germline *EPCAM* gene deletion, which was discovered during ovarian tumor surgery.

## Case presentation

A 59-year-old woman visited her primary care physician for discomfort in the lower right abdomen. As no abnormalities were identified from gastroscopy or colonoscopy, her physician scheduled routine follow-up. Three months later, she again visited her physician complaining of hypertension. Serum carcinoembryonic antigen (CEA) level was elevated at 23 ng/mL (normal range, 0–5 ng/mL), and computed tomography (CT) showed a 65-mm tumor in the right ovary. She was referred to our hospital for further examination and treatment.

**Fig. 1:**
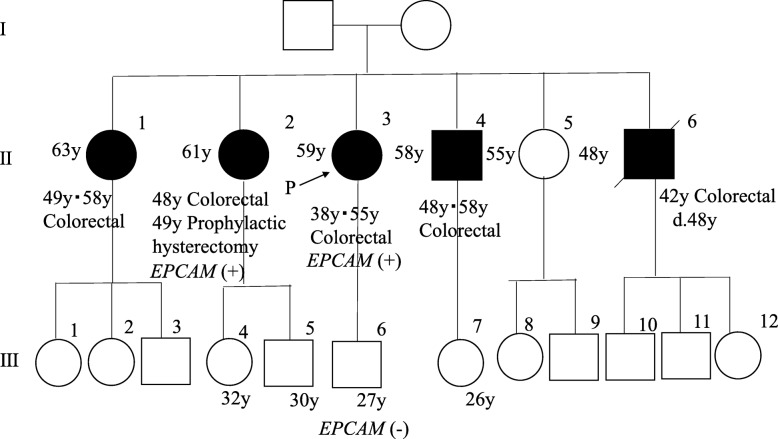
Family pedigree. Four siblings (II-1, − 2, − 4, − 6) had a history of colorectal cancer, and two siblings (II-1, − 4) had a history of multiple colorectal cancers. An older sister (II-2) received a diagnosis of LS attributable to an *EPCAM* deletion and underwent prophylactic hysterectomy

She developed transverse colon cancer at 38 years old and underwent transverse colectomy and lymph node dissection. At 55 years old, intramucosal colorectal cancer was diagnosed and endoscopically removed. The patient’s family pedigree is shown in Fig. 1. She had five siblings, four of whom (II-1, − 2, − 4, − 6) had a history of colorectal cancer and two of whom (II-1, − 4) had a history of multiple colorectal cancers. *EPCAM* deletion was identified in an elder sister (II-2) and she underwent prophylactic hysterectomy and bilateral salpingo-oophorectomy in the United States.

**Fig. 2:**
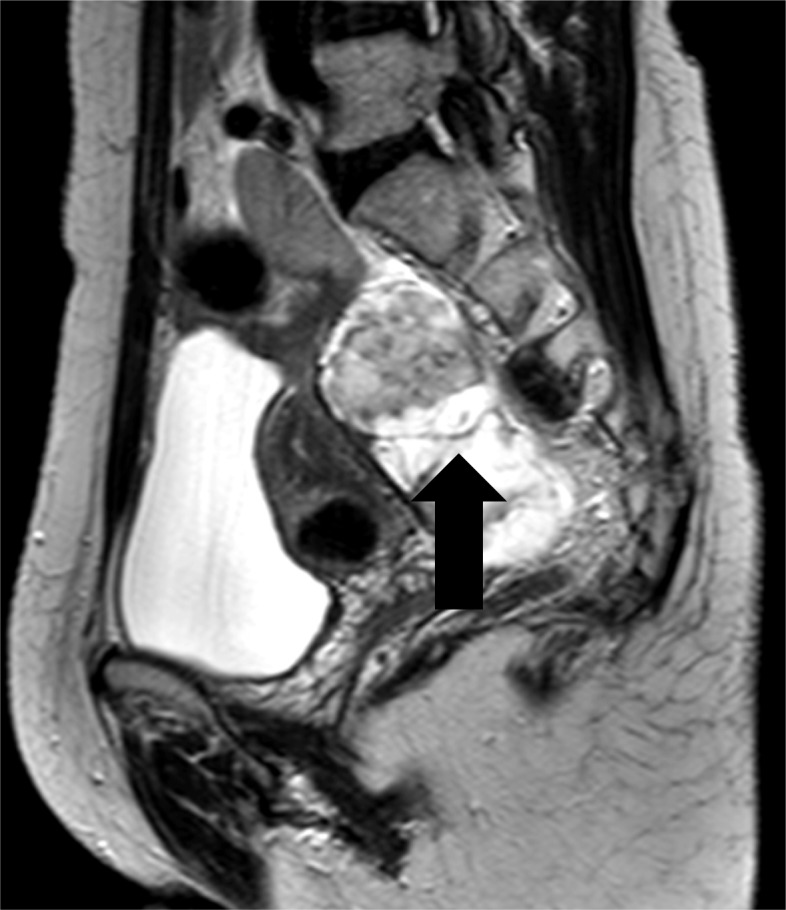
Sagittal plane of the pelvic MRI. T2-weighted MRI of the pelvis reveals a tumor with solid components on the dorsal side of the uterus. The tumor interior shows signal hyperintensity

Pelvic contrast-enhanced magnetic resonance imaging (MRI) revealed a tumor with solid components measuring 50 × 78 × 36 mm on the dorsal side of the uterus (Fig. 2). The interior of the tumor showed signal hyperintensity on T2-weighted imaging. The tumor showed lobular growth but had not infiltrated surrounding areas. Contrast-enhanced CT showed no distant metastases or lymph node metastases. Serum CEA level was elevated at 18 ng/mL. Carbohydrate antigen 19–9 and carbohydrate antigen 125 levels were within normal ranges.

**Fig. 3:**
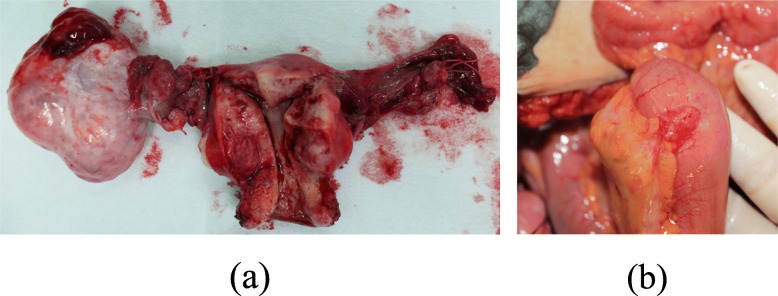
Surgical findings. **a** The right ovarian tumor has a lobular interior, and is primarily mucinous with some solid portions. **b** The small bowel tumor shows papillary growth in the lumen

As the above findings suggested, the tumor could be either primary or secondary ovarian cancer. Therefore, we decided to perform surgery to allow for pathological examination. We performed total abdominal hysterectomy, bilateral salpingo-oophorectomy, and partial omentectomy. There was a fist-sized, lobular, cystic tumor in the right adnexa. The right ovarian tumor measured 8.5 cm in diameter, had a lobular interior, and was primarily mucinous with some solid portions (Fig. 3a). Ascites was absent, and the uterus and left adnexa appeared normal. During surgery, we found a cherry-sized tumor in the ileum, located on the 20-cm mouth side from the ileocecum. Subsequent ileal resection was performed. The ileum tumor measured 2.5 cm in diameter and showed papillary growth in the lumen (Fig. 3b).

**Fig. 4:**
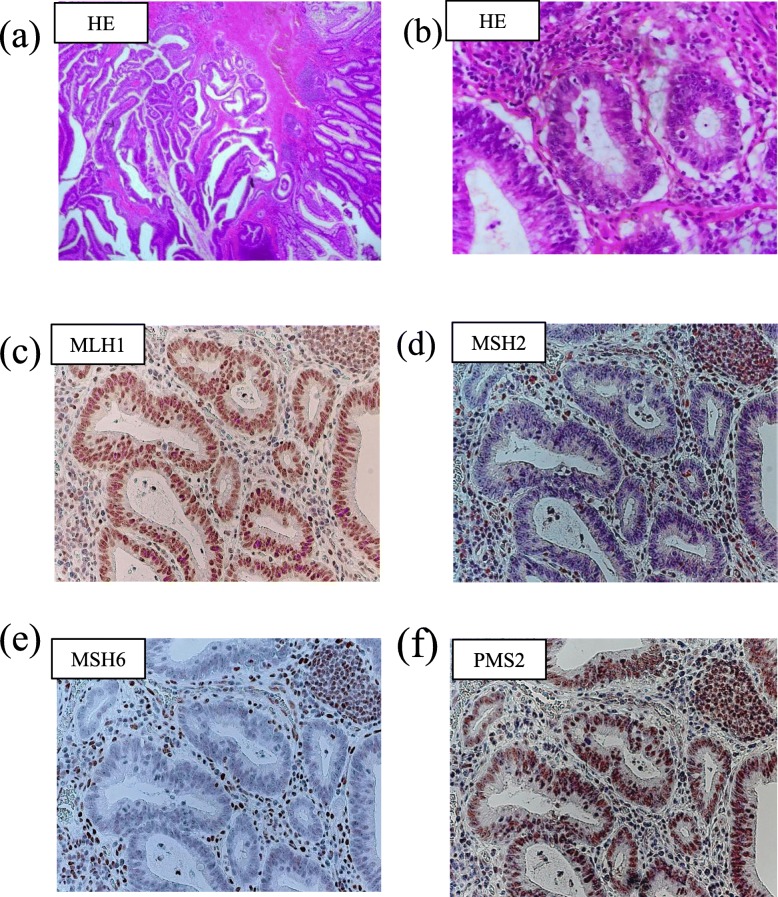
Pathological examination of the small bowel tumor. Hematoxylin and eosin staining (**a**) 100× magnification and (**b**) 200× magnification. Atypical columnar cells growing in irregular/fused duct patterns are present. Immunohistochemical staining of MMR proteins (**c**) MLH1, (**d**) MSH2, (**e**) MSH6, (**f**) PMS2. The expression of MSH2 and MSH6 are decreased

Histopathological examination revealed mucinous adenocarcinoma of the ileum. Atypical columnar cells growing in irregular/fused duct patterns were present in the ileum. Immunohistochemical staining of the tumor cells was negative for keratin 7 and keratin 20. The tumor had infiltrated to the subserosa and was partially exposed to the serosa (Fig. 4a, b). The ovarian tumor showed the same pathological features. Finally, the patient was diagnosed as having ileum cancer with ovarian metastasis (pT4NXM1, stage IV).

Furthermore, we performed immunohistochemical examination of MMR proteins. While MLH1 and PMS2 nuclear staining remained intact, MSH2 and MSH6 staining was decreased in both the ileum and ovarian tumor (Fig. 4c, d, e, f).

The patient recovered uneventfully and was discharged 10 days after surgery. We referred her to the Department of Gastroenterology of our hospital for adjuvant chemotherapy. She received chemotherapy comprising intravenous oxaliplatin 130 mg/m^2^ on day 1 and oral capecitabine 1000 mg/m^2^ twice daily from days 1 to 14, every 3 weeks (CAPOX regimen). After four courses of CAPOX, the treatment regimen was switched to capecitabine monotherapy because of an oxaliplatin-related adverse event. She received two courses of capecitabine monotherapy, thereby completing initial treatment. Currently, she has remained disease-free for 24 months.

Because her medical and familial histories and immunohistochemical examination of MMR proteins suggested LS, we referred her for genetic counseling in the Department of Medical Genetics of our hospital. Based on genetic information from her older sister, genetic testing was performed with her consent. Multiplex ligation-dependent probe amplification assay showed deletions of *EPCAM* exons 8 and 9, upstream of *MSH2*, confirming LS. Her son (III-6) also underwent genetic testing, but the results were negative.

## Discussion and conclusions

This represents the first report of ileum cancer in a patient with germline *EPCAM* gene deletion, which is a novel cause of LS. The *EPCAM* gene encodes a type I transmembrane glycoprotein and is located 17 kb upstream of *MSH2* on chromosome 2. In most *EPCAM* carriers, germline deletions were found in regions including the polyadenylation site located in the last two exons (8 and 9), which lead to epigenetic silencing of *MSH2* [6]. Because immunohistochemical staining for MMR proteins showed *MSH2* inactivation in the tumor tissue, ileum cancer in the present patient is very likely associated with LS.

Patients with LS have a 1–4% lifetime cumulative incidence of small bowel cancer, about 100-times the lifetime risk in the general population [7]. Sporadic small bowel cancers generally occur in the duodenum, while LS-associated small bowel cancers frequently originate in other areas (43% in duodenum, 37% in jejunum, and 20% in ileum), showing a different distribution [8]. A study of 667 LS patients in the Netherlands found that 3 of 194 patients with *EPCAM* deletions (1.5%) developed duodenal cancer. However, ileum cancer has not previously been reported in *EPCAM* carriers [9].

Few studies have documented the clinical features of *EPCAM* deletion with LS. Evaluating a population of 667 LS patients that included 194 patients with *EPCAM* deletion, Kempers and colleagues concluded that the risk of non-colorectal LS-associated cancers in *EPCAM* deletion carriers was lower than that in four MMR gene variants [9]]. Although the lifetime risk of colorectal cancer was similar at 75% in *EPCAM* deletion carriers and 70% in *MSH2* variants, the lifetime risk of endometrial cancer was 12% in *EPCAM* deletion carriers, but 51% in *MSH2* variants. They suggested that the risk of extra-colonic cancer may be associated with *EPCAM* deletions, depending on whether a deletion affects only the *EPCAM* gene or both *EPCAM* and *MSH2* genes. The present case revealed that ileum cancer was likely associated with LS, which indicates the need to consider small bowel cancer as LS-associated cancer in patients with *EPCAM* deletions.

Careful observation during ovarian tumor surgery allowed us to identify the ileum cancer that would have been difficult to find preoperatively in the patient. As clinical symptoms are frequently vague and non-specific, patients with ileum cancers are often diagnosed at advanced stages [10]. Furthermore, there are currently no effective methods to detect early-stage small bowel cancer in asymptomatic individuals. Video capsule endoscopy (VCE) and double-balloon endoscopy have recently emerged as alternatives to CT and MRI for identifying small bowel cancers. Haanstra and colleagues note that small bowel cancer can be overlooked in VCE [11]. In a prospective study evaluating the significance of VCE in diagnosing small bowel cancer in 200 patients with LS, 2 patients developed small bowel cancer. The disease was detected in one of the patients with VCE but was not found with VCE in the other, in whom stage II disease was diagnosed 7 months after negative VCE. VCE is not recommended for the surveillance of small bowel cancer in LS but is recommended for patients with symptoms suggestive of small bowel cancer [12].

The prognosis of small bowel cancer is poor, with 5-year survival rates of 59.7% in the United States [13] and 48% in Europe [14]. Given the rarity of small bowel cancer, no evidence-based standard treatment has been established. Surgical resection is the most common option for localized tumors. In a retrospective study performed in Japan, median overall survival in 10 patients with stage IV small bowel cancer who underwent surgical resection or local radiotherapy plus chemotherapy was 36.9 months [15], suggesting that a multidisciplinary approach could improve prognosis even in patients with distant metastases. Although the present patient was diagnosed at an advanced stage, complete resection was possible because the lesions were limited to the small bowel and ovary. She was subsequently treated using the CAPOX regimen because chemotherapy for small bowel cancer often includes fluoropyrimidine and platinum combination therapy according to gastric or colorectal cancer [16]. Surgery and adjuvant chemotherapy have kept the patient recurrence-free to date.

We reported here the first case of ileum cancer in a patient with germline *EPCAM* gene deletion, which can cause LS. Fortunately, we could identify ileum cancer that would have been difficult to find preoperatively through careful observation during ovarian tumor surgery and successfully treated the patient with surgical resection and CAPOX chemotherapy. Immunohistochemical staining for MMR proteins indicated the ileum cancer likely occurred as LS-associated cancer. When treating patients with hereditary cancer syndromes including LS, we should keep all associated cancers in mind.

## Data Availability

The data showed in the report is not publicly available due to this containing information that could compromise the privacy of this patient and her family but is available from the corresponding author upon reasonable request.

## Abbreviations

CAPOX: Capecitabine and oxaliplatin
CEA: Carcinoembryonic antigen
CT: Computed tomography
EPCAM: Epithelial cell adhesion molecule gene
LS: Lynch syndrome
MMR: DNA mismatch repair
MRI: Magnetic resonance imaging
VCE: Video capsule endoscopy
